# A study protocol of mobile phone app-based cognitive behaviour training for the prevention of postpartum depression among high-risk mothers

**DOI:** 10.1186/s12889-019-6941-8

**Published:** 2019-06-07

**Authors:** Mei Sun, Siyuan Tang, Jiarui Chen, Ying Li, Wenhui Bai, Virginia Plummer, Louisa Lam, Chunxiang Qin, Wendy M. Cross

**Affiliations:** 1 Xiangya Nursing School, Central South University, 172 Tongzipo Road Yuelu district, Changsha, 410013 Hunan China; 2 Henan Provincial People’s Hospital, Zhengzhou, 450000 China; 3 School of Nursing and Midwifery, Monash University, Melbourne, VIC 3800 Australia; 4 Peninsula Health, Frankston, VIC 3199 Australia; 5 School of Nursing and Healthcare Professions, Federation University, Melbourne, VIC 3806 Australia; 6 Obstetrical Department, The Third Xiangya Hospital, Central South University, Changsha, 410013 China

**Keywords:** Postpartum depression, Cognitive behaviour training, Mobile phone applications, Negative emotion symptoms, Parenting competence

## Abstract

**Background:**

The changes in China’s family planning policy in recent years have led to changes in the age structure of pregnant women, and the prevalence of postpartum depression (PPD) is also on the rise. Cognitive Behaviour Training (CBT) as an effective intervention is widely used for postpartum depression. However, the shortage and health disparities of mental health resources, the stigma of postpartum depression in postpartum women and the need for postpartum recovery and child care prevent postpartum women from seeking traditional face-to-face CBT. Therefore, the purpose of this proposed study is to examine the effect of mobile phone applications (App) based CBT on postpartum depression, anxiety, pressure and parenting sense of competence.

**Methods:**

A double blind, randomized controlled trial will be used in this study to examine the effectiveness of App-based CBT in reducing the prevalence of postpartum depression compared with usual postpartum care in China. A total of 120 participants will be recruited in this study. The intervention consists of a weekly theme module app for continuous six weeks, each module including learning content and assignments. The control group received usual postpartum care content through the App. Outcome measures include postpartum depression, anxiety, pressure and parenting sense of competence at 0-, 3- and 6-month after the intervention.

**Discussion:**

If our intervention is effective, it will provide a time-friendly and unrestricted intervention for the psychological care of perinatal women, which can effectively solve the shortage and unevenness of mental health resources.

**Trial registration:**

Chinese Clinical Trial Registry, ChiCTR1900020735. Registered 15 January 2019.

## Background

Mental health has become a global issue for public health. The global burden of disease 2016 (GBD 2016) confirmed that the major cause of non-fatal burden was mental disorders and substance use disorders, led by depressive disorders [1, 2]. Postpartum depression (PPD) as a disabling but treatable mental disease is one of the most common mental health issues faced by post-natal women. The Diagnostic and Statistical Manual of Mental Disorders, Fifth Edition (DSM-5) defines PPD as a major depressive episode that occurs during pregnancy or within 4 weeks of delivery [3]. This is because the effects of biological factors on women’s emotions are significantly reduced impacted within 4 weeks after birth. However, the pressures of parenthood faced by some postpartum women are difficult to alleviate, and depression during this period continues to have negative consequences, which require medical treatment [4, 5]. Therefore, in the current study focusing on PPD, the postpartum period is defined as one year after birth [6].

According to a report conducted by World Health Organization (WHO) [7], approximately 10% of pregnant women and 13% of women who have just given birth worldwide suffer from mental disorders, mainly depression. A recent meta-analysis of studies from 56 countries showed that the global prevalence of PPD is about 17.7% [8]. The national differences in the prevalence of PPD is mainly reflected in higher prevalence among women in lower-income and middle-income countries [9–11]. Studies in China in recent years have shown that the prevalence of PPD is approximately 25% [12, 13], which is higher than the global average. That may be partly due to the special condition of fertility policy in China. As an effective approach to decelerate the growth of population, the one-child policy has been implemented in China since the 1980s. Now the first generation of ‘only children’ following the implementation of that policy has reached reproductive age. Some research shows that women from one-child families are more likely to have PPD [14, 15]. Moreover, with the recent change of family planning policy in China, many women have more than one child now. This has resulted in the changing age structure of pregnancy women, which is a clear factor for the increased proportion of advanced maternal age (AMA) [16]. This change may consequently increase the prevalence of PPD by increasing pregnancy complications as well other adverse pregnancy outcomes [17, 18].

As one of the most common mental health disorders during the postnatal period, the negative effects of PPD involve many aspects of daily life. These include lower quality of life for women [19], physical and psychological developmental disorders in children [20], and lower marital satisfaction [21]. Psychological interventions including health education, interpersonal psychotherapy (IPT), cognitive behaviour training (CBT) and problem focused therapy have been the main interventions for PPD due to concerns about the adverse effects of pharmacology therapy on breast feeding infants [22]. Among these psychological therapies, CBT is considered to be the most widely used. The effectiveness of CBT in mild to moderate depression has been demonstrated in many studies [23, 24]. Multiple guidelines and expert consensus also recommend CBT as an effective psychological intervention for PPD [25, 26]. Nevertheless, there are still many barriers to the successful implementation of face to face CBT in the postpartum population. First, the shortage and health disparities of mental health resources, for example lack of mental health care workers, hinder the accessibility of mental health services [27]. Second, the stigma of PPD blocks seeking “face-to-face” mental health services, which are particularly prominent in lower-income and middle-income countries [28–30]. Lastly, the challenge of finding time for mothers to participate in the psychological intervention while trying to take care of their babies is also another barrier [27].

With the development of internet technology and mobile health in recent years, Computerised Cognitive Behavioural Therapy (CCBT) as well as health promotion mobile phone applications (App) have been used in many countries for the treatment of depression [31], anxiety [32] and insomnia [33]. In recent years, the continuous development of mobile communication networks and the wide application of smartphones have also provided new ways to realize psychological interventions based on mobile health technologies. It is reported that [34], as of June 2016, the number of mobile internet users in China reached 656 million, and the internet access equipment was further concentrated to the mobile terminal, which laid the foundation for the development of health services based on mobile health technology. CCBT and App-based CBT are easily accepted by postpartum women because of their flexibility in participation time and confidentiality of participant information. And for the providers of mental health services, CCBT and App-based CBT provide a good solution of the shortage of the mental health services resources. However, there is no currently CCBT or App-based CBT for women with PPD. Therefore, the purpose of this proposed study is to examine the effect of App-based CBT on psychosocial outcomes (including postpartum depression, anxiety, pressure and parenting sense of competence).

Based on this, we hypothesize that the App-based CBT will enable mental health services to be delivered to perinatal women in a more convenient, flexible and personalized way so that it can avoid the disadvantages of traditional CBT in terms of accessibility for perinatal women.

## Methods/design

### Design

A double blind, randomized controlled trial (RCT) will be used in this study. All participants who met the inclusion criteria were randomly assigned to the intervention group or control group for a six-week intervention program.

### Participants

This study will be conducted in Changsha city, which has five administrative district. We will randomly select one health center from all the community health centers in each district. Participants will be recruited from five community health centers we selected. Inclusion criteria:

1. has Established maternal health care record in community health centers;
2. 0 days to 6 weeks after delivery;
3. Score of 9–12 on the Edinburgh Postnatal Depression Scale (EPDS);
4. Access to smart phone and able to independently use App;
5. Informed consent and voluntary participation.

Exclusion criteria:

1. Severe mental disorders (current or in the past);
2. History of brain injury, intellectual disability or cognitive disorder; and
3. Undergoing psychotherapy or any other psychological intervention.
4. Patients diagnosed with postpartum depression or other severe mental illness during the study.

### Measures

Measurements will be obtained at baseline (t_o_), immediately after the last intervention (t_1_), 3 months following the intervention (t_2_) and 6 months following the intervention (t_3_). The baseline data is collected when participants are enrolled, and after that, the data collection process will be implanted in the evaluation module of the mobile App. The primary outcome will be depression status. The secondary outcomes will be negative emotion symptoms including depression, anxiety and stress, parenting confidence. These will be measured as follows:

1. *Demographic and Socioeconomic factors:* the information about Risk factors will be collected by demographic questioner including pregnancy and birth history, obstetric complications, delivery mode, baby gender, baby weight, baby health status, feeding pattern, satisfaction with family care, major life events. The socioeconomic factors include maternal age, marital status, history of depression, family history of mental disorder, occupation, medical insurance, education level, family income, and religious belief.
2. *Depression status:* We will use the Edinburgh Postnatal Depression Scale (EPDS) Chinese version to assess participant’s depressive status, as this scale is an effective self-screening tool for PPD. The EPDS to be used in this study was translated by Lee [35] the sensitivity was 82%, the specificity was 86%, the positive predictive value was 44%, and the negative predictive value was 97%. The scale includes 10 items. The recommended threshold is < 9: no depression; 9 is regarded as a probable case; and 13 or more as suspected depression.
3. *Negative emotion symptoms:* We will assess negative emotion symptoms using the Depression, Anxiety and Stress Scale (DASS-21), which is a self-report measure of anxiety, depression and stress levels used in diverse settings developed by Lovibond [36]. The Chinese version of DASS-21 was developed by Taouk ^[40]^ in 2001. This version was tested in Australian-Chinese speaking samples and Hong Kong-Chinese speaking samples, and indicated that the items had been adequately and appropriately translated and adapted. Wen Yi et al. [37] tested DASS-21 in 730 Chinese adults (over 18 years old), the result showed that the Cronbach’s α was 0.912 and the test-retest Pearson correlation coefficient was 0.751.
4. *Parenting competence:* Parenting competence will be assessed using the Chinese version of Parenting Sense of Competence Scale (C-PSOC). The initial Parenting Sense of Competence Scale was an English version developed by Gibaud-Wallston [38]. In 2014 Chinese researcher, Yang Xiao translated it into Chinese and applied to Chinese population. C-PSOC includes efficacy and satisfaction factors and has 17 items. The Cronbach’s coefficient, efficacy factor and satisfaction factor of C-PSOC were 0.82, 0.80 and 0.85 respectively [39].

### Conditions

#### The intervention condition: app-based CBT

App-based CBT is comprehensive App that including investigation and intervention. The investigation section includes four surveys (baseline, immediately after the intervention, three months after the intervention and six months after the intervention). The content of investigation is mentioned in the measures section. Taking into account the needs for women to rest and care for their newborns after giving birth, in order to ensure the effectiveness of the intervention, our research team conducted a survey of the needs of mental health services for postpartum women before determining specific interventions. Based on this result, we developed intervention program. The intervention consists of six modules, participants need to complete one module each week for six consecutive weeks, and each module will takes an average of 30–40 min. Participants will receive a reminder if they haven’t started reading the content of each module for the fourth day after the module is opened. Module 1 (prologue) will help participants to gain a preliminary understanding of postpartum depression. Module 2 (emotion) will help participants understand the negative emotions and identify them. Module 3 (recognition) will assist participants recognize the erroneous thinking. Module 4 (amendment) will provide some strategies for participants to deal with the biased habitual thinking. Module 5 (rebound) will help participants focus on the present life and avoid immersing in negative emotions. Module 6 (remain happiness) will help participants review the techniques of emotion management and getting rid of biased thinking, so that they can keep happy (Table 1).

**Table 1:** The conditions between intervention group and control group

Time	Intervention group	Control group
1 week	Prologue will help participant to understand the postpartum depression a. Using examples to introduce what is postpartum depression, PPD symptoms, and how to differentiate PPD with the normal changes which might happen after delivery b. New progress of the treatment and intervention of PPD c. Skill learning: Self-evaluate the status of PPD	a. Postpartum perineum incision care or caesarean section wounds; b. Explanation of lochia postpartum; c. Prevention of postpartum constipation
2 week	Emotion will help participants a. Understand what negative emotions is. b. Record negative emotions and the corresponding behavioral responses c. Skill learning: think differently	Neonatal feeding (breast feeding or bottle feeding) a. Encourage breastfeeding, and how to breastfeeding correctly. b. Identify situations that require bottle feeding
3 week	Recognition will assist participants understand a. What is the biased habitual thinking? b. What is the relationship between biased habitual thinking and negative emotions? c. Skill learning: identify the biased habitual thinking.	Neonatal care (bathing and touch) a. Time, temperature and supplies of bathing; b. Navel nursing c. Neonatal touch method
4 week	Amendment will provide some strategies for participants to deal with the biased habitual thinking a. Using example to help participants understand what is the consequences of the biased habitual thinking. b. How to divert their attentions from the troublesome thing. c. Skill learning: introspect your thinking.	Postpartum recovery (early training) a. The timing and frequency of getting out of bed b. Intensity of postpartum exercise
5 week	Rebound will help participants focus on the present life and avoid immersing in negative emotions. a. Record the daily life b. Balance your life c. Skill learning: Set short-term goals and long-term goals and	neonatal vaccination a. Knowledge of infant vaccination
6 week	Remain happiness will help participants a. Review the techniques of emotion management and getting rid of biased thinking b. Skill learning: Record the daily happy things	postpartum reproductive health (contraception) a. Choice of contraceptive methods

#### Control condition

Participants in control group will also complete six health education modules which are formulated according to the standard of health management service postpartum and the child health management through the App. It mainly includes the care of postpartum perineum incision and caesarean section wounds, breast or bottle feeding, postpartum recovery, neonatal care (bath and touch), neonatal vaccination and postpartum reproductive health (contraception) (Table 1).

### Sample size

A power analysis was performed for linear regression using University Dusseldorf G*Power 3.1 to determine the sample size necessary for analysis. The main objective of this study is to assess the effectiveness of App-based CBT on PPD high-risk mothers. According to previous studies, an effect size of 0.43 will be adopted [40]. The power analysis based on the conventional significant alpha level of 0.05 and a power of 0.90 using a two-tailed test. We calculate that the sample size required is 48 in each group. Considering a 20% dropout rate, the recruited sample size required in each group is 60.

### Procedure

For the participants who meet the inclusion criteria, the researchers will introduce the research content to them to confirm their willingness to participate in the study. Participants will also be asked whether they understand the benefits and possible risks involved in this study. For participants who agree to join in the study, the researchers will ask them to download the App and researchers will issue an informed consent form and inform participants that they will be randomly assigned to different groups, and all the individuals have the right to consult to researchers. If the participant does not agree to be involved in the study, the researchers will record the reasons for their refusal. The schedule of enrolment, interventions, and assessments are summarized in Table 2. If the intervention was shown to be effective, participants in the control group would receive the intervention immediately after the study was completed.

**Table 2:** The timeline of enrolment, interventions and assessments

	Study period										
	Enrolment	Allocation	Intervention						Follow-up		
Timepoint		t_0_	1st week	2nd week	3rd week	4th week	5th week	6th week	t_1_	t_2_	t_3_
Enrolment:											
eligibility screen	●										
informed consent	●										
allocation		●									
Interventions:											
intervention group			●	●	●	●	●	●			
control group			●	●	●	●	●	●			
Assessments:*											
baseline		●									
immediately after the last intervention									●		
3 months following the intervention										●	
6 months following the intervention											●

*Variables assessed at baseline include demographic factors, socioeconomic factors, depression status, negative emotion symptoms, parenting competence

*Variables assessed at other three times are the same, they were depression status, negative emotion symptoms, parenting competence

### Randomization

The allocation scheme is generated by the random digital program. The whole randomization process is a computer-based algorithm. The starting value will be determined by the current server time and the process of the allocation scheme will be recorded for replication. Randomization will not be stratified and will occur automatically at the completion of the baseline survey by obtaining the next allocation of the generated timeline.

### Blinding

This study will be a double-blind trial. After the baseline survey is completed, the allocation scheme will be generated by a computer program and it will be kept in closed and opaque envelopes and saved by the researchers who have no direct relationship with the project to ensure that the random allocation scheme is hidden in advance. The automatic randomization allocation of the participants will remain blind to the enrolling researcher. Researchers who administered the six-week intervention and analyzed the data were also blind to the grouping. Participants will be informed that this study is to investigate the effectiveness of an App-based intervention program to protected women from PPD, but they will not be informed of anything content related to the specific interventions.

### Analysis

This study used double data entry. Data entrants, analysts, and data custodians do not participate in the intervention process. Analyses will be performed using SPSS (version 20.0; SPSS Inc., Chicago, Ill). All tests of significance will be determined at an alpha level of 0.05. The principle of ITT will be used to handle missing data. We plan to analyse contrasts between intervention and control groups at baseline, post intervention, 3 months and 6-months follow-up.

### Ethical approval

The plan and the design content have been reviewed and approved by Institutional Review Board (IRB) of University (IRB Approval Number: 2018004). Researchers will introduce this program, including the research process, benefits and possible risk of this study, as well as the voluntary nature of the study, to those women who meet inclusion criteria. A written informed consent will also be obtained. Participants have the right to withdraw at any time.

### Risk management

In our study, participants have to complete EPDS at baseline, immediately after the intervention, three months after the intervention, and six months after the intervention. We will refer the participants to psychologist immediately after we found participants who had EPDS scores greater than 12. Each participant is entitled to withdraw from the study during the intervention, when they decided to withdraw from the study, our research team will assess their situation to see if they needed to be transferred to psychologist.

## Discussion

In China, although existing maternal health care regulations clearly propose to provide mental health services during pregnancy and postpartum, most of those services are carried out in hospitals, which reduce their accessibility to some women especially in the postpartum period. There are some health programmes for pregnant women organized by the hospital or community which provide information about mental health, but that is only for pregnant women with no follow-up available. With the advancement of graded diagnosis and treatment of public health, postnatal home visits have become a routine service for community work [41]. Usually, during the first 6 weeks following childbirth community medical professionals will conduct regular visits but the mental health status is not included in the visit. Therefore, developing a systematic, continuous, and accessible psychological intervention is essential. To the best of our knowledge, this proposed study will be the first intervention study to apply CBT to women with PPD through mobile health technology.

The highlight of this study was the application of App-based CBT to postpartum women. Ensuring the effectiveness of intervention is the key to this study. Firstly, the provider of intervention must be professionally trained and supervised [42, 43]. In our research, the intervention program was developed with the participation of psychologists to ensure the professionalism of the CBT program. Secondly, we conducted a survey on the form, frequency, and duration of intervention before the intervention was made to ensure the feasibility of intervention. Within those, the effectiveness and operability of interventions will be guaranteed.

In recent years, the high prevalence of postpartum depression in China and the shortage of mental health service resources make it extremely urgent to effectively intervene postpartum depression. With the development of mobile health technology, this study provides a convenient and effective intervention measure for postpartum depression women. If our intervention program proves to be effective in reducing the prevalence of postpartum depression, this will not only provide convenient and effective intervention measures for postpartum depression and effectively saved mental health service resources, but will also provide evidence-based evidence for interventions based on mobile health technologies.

## Abbreviations

AMA: Advanced maternal age
App: mobile phone applications
CBT: Cognitive behaviour training
CCBT: Computerised cognitive behavioural therapy
C-PSOC: Chinese version of parenting sense of competence scale
DASS-21: Depression, anxiety and stress scale
DSM-5: The diagnostic and statistical manual of mental disorders, fifth edition
EPDS: Edinburgh postnatal depression scale
GBD 2016: Global burden of disease 2016
IPT: Interpersonal psychotherapy
PPD: Postpartum depression
RCT: Randomized controlled trial
WHO: World health organization
